# 

**Published:** 1843-04

**Authors:** 


### BOOKS RECEIVED FOR REVIEW.

ENGLISH.

1. A Practical Treatise on the Teeth, showing the causes of their destruction, and the means of their preservation, with Plates. Third Edition. By W. Robertson. London, 1842. 8vo, pp. 224.

2. The History, Diagnosis, and Treatment of Typhoid and of Typhus Fever, &c. By Elisha Bartlett, M.D., Professor of Medicine in Transylvania University. Philadelphia, 1842. 8vo, pp. 393.

3. The New England Quarterly Journal of Medicine and Surgery, Editors C. E. Ware, M.D., S. Porkman, M.D. Nos. I and II, July and October, 1842.—Boston. [*These are the first numbers of a new Journal of much promise, patronised by the head of the profession in Boston. Supported by such enlightened men, it cannot fail to prosper.*]

4. Physician for Ships; containing medical advice for seamen, &c. Third Edition. By Usher Parsons, M.D., &c.—Boston, 1842. 8vo, pp. 216.

5. The Physical Diagnosis of Diseases of the Lungs. By W. H. Walshe, M.D., &c.—London, 1843. 8vo, pp. 307.

6. Lectures on Animal Physiology; or the physical condition of man as regards health and disease. By B. T. Lowne, esq. —London, 1842. 8vo, pp. 101.

7. Chemical Manipulation: being instructions to Students in Chemistry, &c. By M. Faraday, D.C.L. F.R.S., &c.—London, 1842. 8vo, pp. 664.

8. Thermal Comfort; or Popular Hints for preservation against Colds, Coughs, and Consumption. By Sir Geo. Lefevre, M.D. &c.—London, 1843. 8vo, pp. 81. 1s.

9. An Exposition of the Pathology and Treatment of Tubercular Phthisis. By S. Flood, M.R.C.S.—London, 1842. small 8vo, pp. 82.

10. The Cyclopædia of Practical Surgery, Part XII. Fracture—Gastro-Hysterotomy. January, 1843. 5s.

11. On a peculiar Development of the Bones of the Forearm. By John Gardner, Surgeon.—London, 1843. 8vo, pp. 8.

12. Experimental and Practical Researches on Inflammation, and on the Origin and Nature of Tubercles of the Lungs. By W. Addison, F.L.S.—London, 1843. 8vo, pp. 76. 5s.

13. Elements of Electro-Metallurgy. By Alfred Smee, F.R.S. &c. Second Edit.—London, 1843. 8vo, pp. 338. 10s. 6d.

14. A System of Clinical Medicine. By R. J. Graves, M.D., M.R.I.A., &c.—Dublin, 1843, 8vo, pp. 937. 18s.

15. Transactions of the Medical and Physical Society of Bombay, for the Year 1841.—Bombay, 1841. 8vo, pp. 156.

16. Outlines of Pathology and Practice of Medicine. By W. P. Alison, M.D., &c. Professor of Medicine in the University of Edinburgh. Parts I. & II.—Edinb. 1843. 8vo, pp. 499. 12s.

17. Observations on the principal Medical Institutions and Practice of France, Italy, Germany, &c. By Edwin Lee, M.R.C.S. 2d. Edit.—Lond. 1842. 8vo, pp. 269. 7s.6d.

18. Two Lectures on the Defective Arrangements in large Towns to secure the Health and Comfort of the Inhabitants. By H. Sandwith, M.D.—London, 1843. 8vo, pp. 55. 1s.

19. Views upon the Statics of the Human Chest, Animal Heat, and Determination of Blood to the Head. By Julius Jeffreys, F.R.S.—London, 1843. 8vo, pp. 233. 6s.

20. Observations on the Extraction of Teeth. By J. C. Clendon, Surgeon-Dentist. —London, 1843. 12mo, pp. 80. 3s. 6d.

21. Criminal Jurisprudence considered in relation to Organization. By M. B. Sampson. Second Edition, with Additions. —London, 1843. 8vo, pp. 147. 5s.

22. Interment and Disinterment; or a further Exposition of the Practices pursued in the metropolitan places of Sepulture, &c. By G. A. Walker, Surgeon.—London, 1843. 8vo, pp. 28.

23. Pharmacologia; being an extended Inquiry into the Operations of Medicinal Bodies, &c. By J. A. Paris, M.D. F.R.S. &c. 9th Edit.—Lond. 1843. 8vo, pp. 622. 20s.

24. Elements of Materia Medica and Therapeutics. By A. T. Thomson, M.D., F.L.S., &c. Third Edition.—Lond. 1843. 8vo, pp. 1232. 31s. 6d.

25. Treatment of the Diseases of the Eye by means of Prussic Acid Vapour, &c. By A. Turnbull, M.D.—Lond. 1843. 8vo, pp. 89. 2s. 6d.

26. Fourth Annual Report of the Registrar-General of Births, Deaths, and Marriages in England, pending 1842.—Lond. 1842. Fol. 228.

27. A Treatise on Diet, comprising the Natural History, &c. By W. Davidson, M.D. —London, 1843. 8vo, 383. 6s.

28. The Causes, Nature, and Treatment of Acute Hydrocephalus, or Water in the Head. A Prize Essay. By J. R. Bennett, M.D.—London, 1843. 8vo, pp. 248. 8s.

29. Popular Cyclopædia of Natural Science. Part III, Mechanical Philosophy.—London, 1843. 8vo, pp. 313.

30. The Physiological Anatomy and Physiology of Man. By R. B. Todd, M.D., F.R.S., and W. Bowman, F.R.S. Part I.—London, 1843. 8vo, pp. 200. 7s. 6d.

31. The Hunterian Oration, delivered at the Royal College of Surgeons on the 14th Feb. 1843. By James M. Arnott.—London, 1843. 8vo, pp. 37.

32. A new Theory and Treatment of Disease, founded on Natural Principles. By John Tinnion, M.D., Ayr.—Edin. 1843. 8vo, pp. 35.

33. Derangements, primary and reflex, of the Organs of Digestion; with an Addition. By R. Dick, M.D.—Edin. 1843. 8vo, pp. 389. 7s. 6d.

FOREIGN.

1. Praxeos Medicæ Universæ Præcepta. Auctore Josepho Frank Joannis Petri Filio, &c., &c. Part iii, Vol. ii, Sect. i, continens doctrinam de morbis tubi intestinalis, quam exposuit, F.A. B. Puchelt, M.D. &c. —Lipsiæ, 1842. 8vo, pp. 786.

2. Mémoire sur l'Emploi des Caustiques dans quelques maladies de l'Urètre. Par le Dr. Civiale.—Paris, 1842. 8vo, pp. 59. (not published.)

3. Mémoire sur l'Anatomie pathologique des rétrécissemens de l'Urètre. Par le Dr. Civiale.—Paris, 1842. 8vo, pp. 74, (not published.)

4. Die Krankheiten des höheren Alten und ihre Heilung: dargestellt Von Dr. C. Canstatt. Zwei Bänder.—Erlangen, 1839. 8vo, pp. 268, 421.

5. Die Specille Pathologie und Therapie. Von Dr. C. Canstatt. Erster und Dritter Band.—Erlangen, 1841-2. 8vo, pp. 331, 764.

6. Pharmacopœa Austriaca. Editio quarta, emendation. — Vendibona, 1836. 8vo, pp. 196.

7. Proeve van een wijsgeerig en zielkundig onderzoek omtrent het regt in algemeen en het strafregt in het bijzonder. Door H. J. Schouten, M.D.—Amsterdam, 1841. 8vo, pp. 338.

LIBRARY OF THE

Orleans Parish Medical Society

### INDEX TO VOL. XV.

OF THE

### BRITISH AND FOREIGN MEDICAL REVIEW.

PAGE

Abercrombie, Dr., account of . 20
Abscess, hepatic, cured by puncture 246
Abscesses, M. D'Arcet on multiple 131
Alison, Dr., his practice of medicine 533
Amaurosis, asthenic, cured by spectacles . . . . . . 246
America, Mr. Combe's notes on . 52
Amputation, different modes of performing . . . . . 191
Anatomy, morbid, treatises on . 83
Ancell, Mr., singular case of tumour 153
Ancients, on the physiology of . 439
Aneurism of the aorta, case of . 148
on a variety of false . 155
Apoplexy, pulmonary . . 88
abdominal, in children . 248
Army, medical statistical reports of 1
Army and navy, relative health of . 1-18
Arnold, Dr., on vomiting . . 61
Arnott, Mr., his Hunterian oration 536
Arsenic, new processes for detecting 251
Asphyxia from coal-gas . . 252
Asthma, on quinine in . . . 239
Asylum, lunatic, music in . . 282
Barlow, Rev. Mr., on intellectual philosophy and physiology . . 535
Barrier, M., on children's diseases . 320
Baumès, M., on diseases of the skin 372
Bennett, Dr., on hydrocephalus . 539
Berton, M., on children's diseases . 320
Binns, Dr., his anatomy of sleep . 184
Black, Dr., his retrospective address 228
Bladder, Mr. Coulson on diseased . 228
Blood, on the pus-like globules of . 234
Body and mind, reciprocal influence of 413
Bonnet, M., new mode of couching . 245
Books received for review . 284-577
Bowman and Todd, their anatomy, &c. 537
Brain, on the decussation of fibres in 231
Bright's disease, morbid anatomy of 100
Bronchi, dilatation of . . 85
tuberculous disease of . 86
Budd, Dr., on symmetrical disease . 149
Budge, Dr. on vomiting . . 61
Burgess, Dr., on diseases of the skin 372
Burial-grounds, effects of, on health 513
Burmese empire, medical reports on 1
Burns, ulceration of the duodenum in 155
Calculus, lachrymal, analysis of . 239
Calculi, vesical, treatise on . . 537

PAGE

Calculus, gravel and gout. Dr. Jones on 500
Cancer of the lungs, treatises on . 430
Carpenter, Dr., on vitality . . 134
on cells . . 259
Cataract, new mode of extracting . 245
Cazenave and Schedel on the skin . 372
Cells, on the origin and function of . 259
Cemeteries, on the formation of . 513
Cerebral diseases of children . 324
Ceylon, medical reports on . . 1
Chadwick's, Mr., sanatory report 285-328
Chemical analysis, M. Parnell on . 531
manipulation, Mr. Faraday's 538
Children, treatises on the diseases of 320
Dr. West on the pneumonia of 543
Civiale, M., on diseases of the urethra 346
Clark, Sir James, on medical reform 529
Coal-gas, asphyxia from . . 252
Combe, Mr., his notes on America . 52
Compression of the brain, Mr. Guthrie on . . . 169
Concussion of ditto, Mr. Guthrie on 163
Conolly, Dr., his treatment of the insane . . . . . . 25
his Hanwell report . 218
Conscience, effects of insanity on . 418
Consumption, treatises on . . 540
Cook, Dr., on consumption . ib.
Coryza, an important disease in infants 327
Coulson, Mr., on diseases of the bladder 228
Crichton, Sir Alex. his commentaries 459
Croup, history of an epidemic . 249
M. Berton on . . 326
Curling, Mr., on ulceration of duodenum . . . . 155
Cyanosis, case of, by Dr. Walshe . 147
Dalrymple, Mr., on the placenta . 148
Death, approaching, announcement of 424
Deaths, violent, statistics of . . 79
Deformity, its prevalence in factories 308
Dermis, inflammation of . . 397
Devergie, M., on the detection of arsenic . . . . 251
Dickson, Dr., his "Fallacies" . 124
Disarticulations, M. Lisfranc on . 43
Disease, a new theory of . . 540
Dropsy, renal, M. Rayer on . 484
Eclipse, effect of, on animals . 231
Eczema of the scalp . . 115
Edinburgh lunatic asylum, music in 282

PAGE
Education, effects of . . 416
Elbow-joint, excision of . . 193
Emetics, medicinal . . 78
Emphysema of the lungs . . 86
Electro-metallurgy, Mr. Smee's . 541
Epilepsy, on the nature and causes of 455
England, Dr. Marx's recollections of 19
Erichsen, Mr., on diseases of the scalp 114
Eruptions, pustular, artificial . 390
Exostoses, non-venereal . . 51
Expectant treatment . . 406
Eye, extirpation of . . 244
Turnbull on diseases of . 494
Faraday, Mr., on chemical manipulation . . . 538
Farr, Mr., his statistical reports . 75
Favus of the scalp . . . 121
Faye, Dr., on the vesiculæ seminales 346
Fergusson, Mr., his practical surgery 188
Fever, history of, in various climates 4
prevalence of, among soldiers and sailors . . 9
on the causes of . . 335
the extreme expensiveness of to towns . . 340
Fibrin, on the composition of . 229
on the structure of . . 234
Fistula, M. Lisfranc on . . 42
Fletcher, Dr., his pathology . 367
Flood, Mr., on consumption . 540
Foville, M., on the base of the brain 231
Forget, M., on epidemic meningitis 236
Fracture of the skull, Mr. Guthrie on 173
Fractures of lower end of radius . 242
Freezing, revival after . . 231
Ganglia of the nerves . . 232
Gardner, Mr., his Great Physician . 228
Glands, labial, Sebastian on . 227
Gobee, Dr., his medical contributions 399
Gout, gravel, and calculus, Dr. Jones on 500
Graves, formation of . . 520
Gravel, calculus, and gout, Dr. Jones on 500
Green, Dr. H., on tubercle in the brain . . . 152
Greenhill, Dr., his Theophilus . 439
Gulliver, Mr., on fibrin . . 234
on pus-like globules . ib.
Gully, Dr., on the simple treatment of disease . . . 405
Guthrie, Mr., on injuries of the head 161
Hanwell, Dr. Marx's account of . 25
Dr. Conolly's report of . 218
Hasse, Dr., his morbid anatomy . 83
Health of the poor, Mr. Chadwick on 328
of different classes of society 336
Board of, Mr. Chadwick on . 342
Heart, malformation of . . 154
and arteries, on the function of 459
Hematuria . . . 477-8
endemic, in the Mauritius 482
Hemorrhage, renal . . 478
uterine . . 528

PAGE
Hernia, M. Lisfranc on . . 37
Herophilus, Professor Marx's account of . . . 106
Herpes . . . 387
Hoskins, Dr., on vesical calculi . 537
Houses of the poor, very unwholesome 334
Hunterian Museum, catalogue of . 195
Oration, Mr. Arnott's . 536
Hydrocephalus, M. Berton on . 324
acute, Dr. Bennett on 539
Hydrometer . . . 105
Hydronephrosis, M. Rayer on . 484
Hypertrophy of the kidneys . 483
Impetigo . . . 389
of the scalp . . 117
Indigestion, Dr. W. Philip on . 524
Inoculation of measles . . 235
Insane, treatment of, at Hanwell . 25
Insanity, religious . . . 242
Sir A. Crichton on . 467
Interment, effects of, on health . 513
Mr. Walker on . . 542
Intestines, inflammation of mucous membrane of . . . 94
condition of, in typhus . 95
Iris, on the erectility of . . 233
Jackson, Mr., on the spleen . 455
Joints, white swellings of . . 48
Jones, Dr. B., on gravel and calculus 500
Journals, British, list of papers in 256-575
Kerst, Dr., his surgical miscellanies 399
Kidneys, M. Rayer on diseases of . 470
hypertrophy of . . 483
diseases of the bloodvessels of 486
fatty . . . 487
tuberculous disease of . 488
cancer of . . . 490
entozoa in . . . 491
malformations of . . 492
Kleffens, Dr. Van, on cancer of the lungs . . . 430
Lallemand, M. on spermatic discharges 346
Lee, Dr., his clinical midwifery . 226
Lefevre's, Sir George, thermal comfort 541
Lepra and psoriasis . . . 395
Lichen . . . . 392
Liebig, Professor, his medical doctrines 501
Lisfranc, M., his clinical surgery . 30
Liston, Mr., variety of false aneurism 155
Liver, diseases of, in the army and navy 16
abscess of, cured by puncture . 246
Lungs, diseases of, in the army and navy 11
Dr. Walshe on diseases of . 223
cancer of . . . 430
Lupus, on the treatment of . . 36
M'Cormac, Dr., his methodus medendi . . . 533
Mackinnon, Mr., on the health of towns . . . 513
Malgaigne, M., on capital operations 240
Malleolus, excision of . . 400
Man, the natural history of . 180

PAGE
Manufactures, influence of, on health 285
Marx, Dr., his recollections of England 19
his Herophilus . . 106
Measles, inoculation of . . 235
Medicine, Dr. M'Cormac's practice of 533
Dr. Alison's practice of . ib.
Memory, in relation to insanity . 419
Meningitis, cerebro-spinal, epidemic 236
Mental decay . . . . 420
Midwifery, Dr. Lee's clinical . 226
Dr. Ramsbotham's . 527
Mind and body, reciprocal influence of 413
Navy, reports on the health of . 1
and army, relative health of . ib.
cause of the improved health in 11
absence of consumption in . ib.
Nævi, on the treatment of . . 239
Nephrotomy, M. Rayer on . . 475
Nerves, on new ganglia of . . 232
Newnham, Mr., on the body and mind 413
Norway, statistics of the medical profession in . . . . . 282
Operations, capital, results of . 240
Ophthalmia, nervous . . . 32
Original papers in the British journals . . . . . 256-575
Owen, Mr., his Hunterian catalogue 195
Paget, Mr., on symmetrical disease . 149
his microscopic results . 542
Pancreas, case of disease of . 157
Parnell, Mr., on chemical analysis . 531
Passions, effects of . . . 417
Pathology, Dr. Fletcher's . . 367
Dr. Alison's . . 533
Payne, Dr., on vitality . . 134
Pelviotomy . . . 247
Pemphigus . . . . . 385
Pharmacopœia, the prescriber's . 227
Philip, Dr. W., on indigestion . 524
Phillips, Mr., on tumour in the neck 155
Phthisis, morbid anatomy of . . 89
its prevalence in factories 310
Physicians, English, estimate of . 27
Physiology and intellectual philosophy 535
Physiology and anatomy of man . 537
Pitting in smallpox, mode of preventing 383
Placenta, Mr. Dalrymple on . 148
Pneumonia, morbid anatomy of . 88
Pneumonia of children, Dr. West on 543
Prichard, Dr., natural history of man 180
Pyelitis, M. Rayer on . . 470
Prolapsus of the umbilical cord . 314
Prurigo . . . . 392
Pulmonary diseases, comparative rarity of in the navy . . . 11
Pus, its poisonous effects on the blood 131
Putrid meat, poisoning by . . 238
Quinine in typhus fever . . 235
Quinine, new method of administering 236
Quinine in asthma . . . 239
Radius, fractures of lower end of . 242
Ramsbotham, Dr., his midwifery . 527

PAGE
Ranula, composition of the fluid of . 236
Rayer, M. on diseases of the kidneys 470
Reform, medical, Sir James Clark on 529
Remak, Dr., on new ganglia . 232
Renal fistulæ . . . . . 477
Renal hemorrhage . . . 478
Report of the Registrar-general . 74
Revulsive treatment . . 407
Rib, removal of a . . 245
Richter, Dr., his medical contributions 405
Riecke, Dr., on burial-grounds . 513
Rokitansky, Dr., his morbid anatomy 83
Rubeola and scarlatina, diagnosis of 382
Rupia . . . . . 386
Saxtorph, Dr., on prolapsus of the cord 314
Scabies . . . . . 397
Scalp, Mr. Erichsen on disease of . 114
Scarlatina and rubeola, diagnosis of 382
Scharling, Dr., on vesical calculi . 537
Sebastian, Prof., on the labial glands 227
Skin, treatises on diseases of . 372
anatomy and physiology of . 378
Skin-diseases, classifications of . 374-5
Skull, great loss of, from trepanning 400
Sleep, the anatomy of . . 184
how procurable . . 187
Smee, Mr., his elements of electro-metallurgy . . . 541
Spermatic diseases, M. Lallemand on 346
Spermatozoa, description of . . 353
Spinal cord, acute inflammation of 403
Spleen, on the functions of . 455
Spooner and Smart, their retrospective address . . . 228
State medicine, encyclopædia of . 225
Sternum, necrosis of . . 399
Stokes, Dr., on cancer of the lungs 430
Stomach, inflammation of . 93
softening of . . ib.
Strabismus, M. Valpeau on . 290
Strophulus . . . . 301
Suicide, statistics of . . 76
Surgery, clinical, M. Lisfranc's . 30
Sydenham Society, prospectus of . 573
Symmetrical diseases . . . 149
Syphilis, mode of preventing . 282
Syphilis, on its treatment without mercury . . . . 468
Taylor, Dr., his tour among factories 285
Temperament, effects of . . 425
Thackrah, Mr., on the effects of trades on health . . . 285
Theophilus on the human body . 439
Thomson, Dr., account of . . 20
Thermal comfort, Sir G. Lefevre on 541
Tinnion, Dr., his new theory of disease 540
Tongue, hairs growing on . . 235
Todd and Bowman, their anatomy 537
Trachea, dilatation of . . 84
case of stricture of . 153
Trades, the effects of, on health . 285
Transactions, medico-chirurgical . 147

PAGE

Tubercles in the brain of children . 152

Tubercles, relative frequency of, in organs . . . . . 236

Tulloch Major, his army reports . 1

Tumours, on the extirpation of . 32

Tumours, singular case of . . 153

in the neck . . 155

Tumours, malignant, diagnosis of . 191

Turnbull, Dr., on the eye . . 494

Turkey, state of medicine in . 282

Typhus, treatment of with Quinine . 235

Typhus fever, Sir A. Crichton on . 462

Ulcers, simple atonic, treatment of . 44

Ulcers, intestinal, Rokitansky on . 97

Ulcers of the legs, treatise on . 226

Ure, Dr., his philosophy of manufactures . . . . . 285

Urethra, M. Civiale on diseases of . 346

Urine, its relations to disease . 501

Uterus, muscularity of . . 527

Vaccination and revaccination, on . 245

Variola, treatment of . . 383

PAGE
Velpeau, M., on strabismus . 240
Vesiculæ seminales, inflammation of 103
treatise on . 346
Villermé, Dr., on the effects of trades 285
Vitality, Drs. Paine and Carpenter on 134
Vomiting, pathology and curative effects of . . . 61
causes of, in man . 64
physiological effects of . 69
therapeutic effects of . 70
Vrolik, Dr., his morbid anatomy . 83
Walker, Mr., on interment . 542
West, Dr., on the pneumonia of children . . . 543
Walshe, Dr., on cyanosis . . 147
on cancer of the lungs 430
Wilson, Dr. his navy reports . 1
Wilson, Mr., on diseases of the skin 372
Worms, singular case of . . 248
Walshe, Dr., on the diseases of the lungs . . . 223
Yacht, a medical, suggestion of . 15

END OF VOL. XV.

C. ADLARD, PRINTER, BARTHOLOMEW CLOSE.

